# Physicochemical Properties of Defatted Rambutan (*Nephelium lappaceum*) Seed Flour after Alkaline Treatment

**DOI:** 10.3390/molecules21040364

**Published:** 2016-03-31

**Authors:** Jirawat Eiamwat, Sorada Wanlapa, Sukit Kampruengdet

**Affiliations:** Food Technology Department, Thailand Institute of Scientific and Technological Research, 35 Moo 3, Technopolis, Khlong Luang, Pathum Thani 12120, Thailand; sorada@tistr.or.th (S.W.); sukit@tistr.or.th (S.K.)

**Keywords:** Rambutan (*Nephelium lappaceum*), flour, physicochemical properties, alkali treatment

## Abstract

Rambutan seeds were subjected to SC-CO_2_ extraction at 35 MPa, 45 °C to obtain defatted rambutan seed flour. Its physicochemical properties before and after treatment with alakali solution using 0.075 N NaOH were investigated. Alkali-treated flour had a significant increment in bulk density, swelling power, water adsorption capacity, emulsion capacity and stability but a reduction in turbidity, solubility and oil absorption capacity. Pasting measurements showed peak viscosity, breakdown, setback and final viscosity increased significantly for the alkali-treated flour, while pasting temperature decreased. The alkaline treatment decreased the least gelation concentration, but increased the apparent viscosity.

## 1. Introduction

Rambutan (*Nephelium lappaceum*) seeds, a fruit waste, contain high amounts of fat (14%–41%) and carbohydrate (28%–46%) [1,2,3]. Although largely underutilized, they could be a useful alternative source for vegetable fat and converted into flour and starch as food ingredients. During recent years, an increased interest in the study of fat from rambutan seeds has been focused mainly on its physicochemical properties [1,2,3]. These investigations suggest possibilities of rambutan seed fat in confectionary products. However, there is a lack of information on flour derived from rambutan seeds for exploiting its potential use in human food products.

Defatting of rambutan seeds has been performed using hexane as solvent [1,2,3], but toxic solvent residues are undesirable and of concern for food applications. Supercritical carbon dioxide (SC-CO_2_) has been proved an effective solvent in the extraction of several seed oils [4] and is acceptable for use in the food processing industry [5].

Aqueous alkali, such as sodium hydroxide (NaOH), is commonly used in the production of starches in the food industry to alter physicochemical properties of starches from different botanical sources. For instance, the alkaline treatment affects the characteristics of sago, potato and corn starches depending on steeping time [6].

In our previous study we have applied SC-CO_2_ for extracting fat and fractionating the oil from rambutan seeds [7,8]. We reported that defatted rambutan seed flour had nutritional potential compared to commercial all-purpose wheat flour [7]. An understanding of the physicochemical properties of the defatted rambutan flour is important for optimizing any potential food industry applications. In the present study, our objectives were: (i) to characterize the physicochemical properties of defatted rambutan seed flour and (ii) to determine the effect of alkali treatment on the flour properties.

## 2. Results and Discussion

In the SC-CO_2_ extraction at 35 MPa, 45 °C, about 30.6% of fat was extracted from rambutan seeds and the yield of the defatted material varied from approximately 63.2% to 65.0%. Table 1 shows chemical composition of defatted rambutan seed flour, before and after alkali treatment. The alkali solution could partially remove protein, fat and amylose contents with reduction percentages of 9.1%, 24.9% and 6.0%, respectively. The ash content of alkali-treated flour was higher than that of untreated flour (*p* < 0.05). The percentage yield of treated flour after the washing process was an average of 60.4%. From this result, the treatment with 0.075 N NaOH for 4 h was less effective in removing protein and fat from the defatted flour.

The Hunter color parameters, water activity and turbidity for untreated and alkali-treated deftatted rambutan seed flours are presented in Table 2. Untreated flour showed a higher value of lightness and lower value of chroma compared to the treated flour. The color values indicated that alkali-treated flour was darker white with increased yellowness. It is possible that alkali-treated flour contained some absorbed material, although some of the soluble components were removed during water washing, resulting in its higher ash content (Table 1). The treated flour showed a lower turbidity value than did untreated flour. This result might be due to lower protein, fat and amylose contents (Table 1), decreasing the light absorption.

Table 3 shows bulk density, water and oil absorption capacity values for untreated and alkali-treated defatted rambutan seed flours. A greater bulk density for alkali-treated flour indicated a better packing than untreated flour. For absorption capacities, alkali-treated flour showed a greater water absorption but a lower oil absorption than untreated flour. This can be explained by the presence of Na^+^ ions adsorbed in the flour’s internal structure, which would increase its hydrophilic tendencies by electrostatic interaction with the hydroxyl groups of water [6]. As a result, more amounts of water could access the structure, increasing the water absorption. A higher oil absorption was attributed to a physical entrapment of oil associated with protein [9].

**Figure 1:**
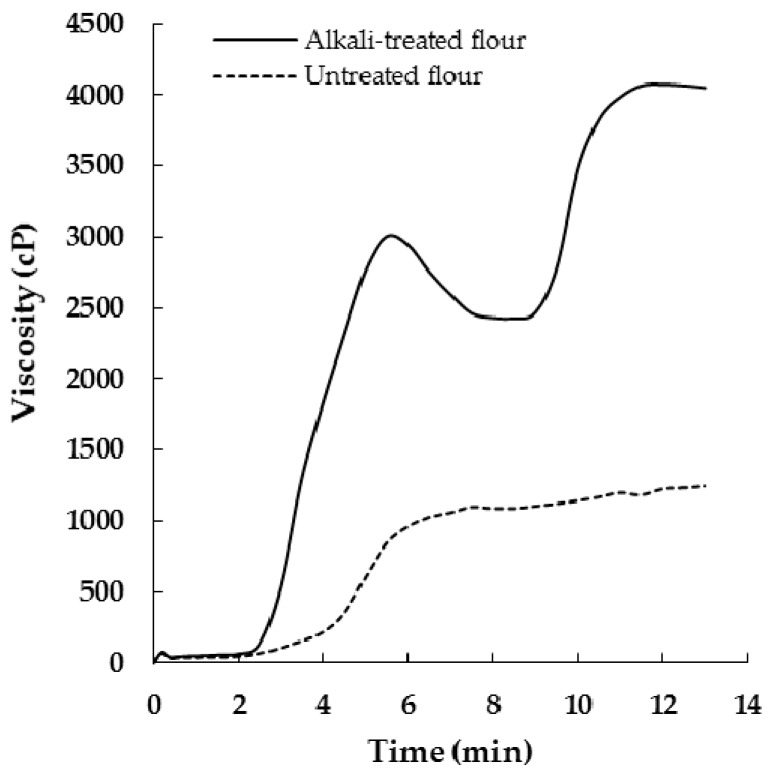
Viscosity of untreated and alkali-treated defatted rambutan seed flours.

Pasting profiles of untreated and alkali-treated defatted rambutan seed flours analyzed using RVA are shown in Figure 1.

The corresponding data are presented in Table 4. Untreated flour displayed a very slow rise in viscosity to a constant 95 °C, followed by a fairly constant viscosity until the end of RVA run. A higher pasting temperature (89 °C) but lower peak viscosity (1056 cP), breakdown (86 cP), final viscosity (1244 cP) and setback (273 cP) were obtained. The pasting viscosity profile suggested the possible existence of crosslinks, which are more resistant to shear during heating and cooling [10].

From Figure 1, alkali-treated flour displayed a viscosity profile similar to a typical V-type with a lower pasting temperature (68 °C), but a greater peak viscosity (3055 cP), breakdown (647 cP), final viscosity (4050 cP) and setback (1643 cP). The treated flour was less resistant to shear and heat, as evidenced by a significant increase in peak viscosity and breakdown. This could be attributed to the presence of OH^−^ ions that might have increased hydration by weakening the bonding within cross-links [6]. Increase in the viscosity on cooling (a high setback value) for alkali-treated flour could be due to a reassociation tendency [11].

Solubility, swelling power and emulsion properties for untreated and alkali-treated defatted rambutan seed flours are given in Table 5. Untreated flour shows a higher solubility value than did alkali-treated flour, presumably due to some of soluble components that could be present before being removed by alkali treatment. A higher swelling power of alkali-treated flour might be attributed to the weakened bonding within crosslinks, thus allowing the material to swell freely as compared to untreated flour. The increase in emulsion capacity and stability after alkali treatment reflects the presence of alkali ions that enhance the water absorption, pasting and swelling power of the treated flour, which could contribute to these observed results.

Gelation properties observed for untreated and alkali-treated defatted rambutan seed flours at different concentrations (2–16 g/100 mL) are shown in Table 6. Untreated flour began gelling at ≥14 g/100 mL, while treated flour showed complete gelling at ≥8 g/100 mL. The reduction in gelation concentration after alkali treatment was attributed to partial reassociation to form gel [11].

**Figure 2:**
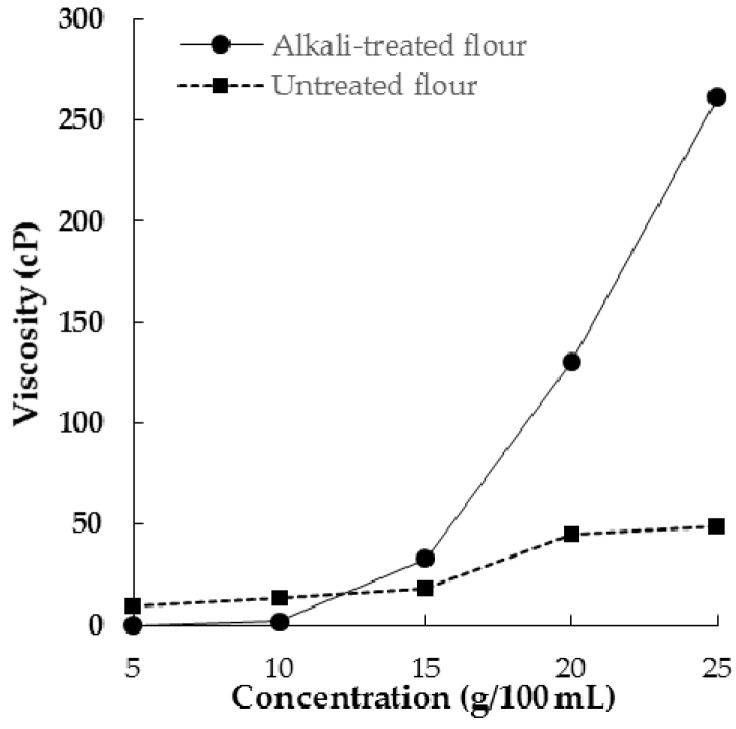
Apparent viscosity of untreated and alkali-treated defatted rambutan seed flours.

Apparent viscosity of untreated and alkali-treated defatted rambutan seed flour at solid concentrations of 5–25 g/100 mL are shown in Figure 2. Untreated flour displayed a first increase in viscosity at 15 g/100 mL and reached a plateau at 25 g/100 mL, whereas a noticeable increase in viscosity of alkali-treated flour was seen at or above 10 g/100 mL. This result agreed with the experiment of least gel concentrations, which was indicative that the alkali ions were mainly responsible for the increase in apparent viscosity.

## 3. Experimental Section

### 3.1. Preparation of Defatted Rambutan Seed Flour

Defatted rambutan seed flour was prepared by SC-CO_2_ extraction of ground rambutan seeds (about 100 g dry basis) at 35 MPa, 45 °C using a Speed SFE instrument (Applied Separations Inc., Allentown, PA, USA) for 44 h. After grinding the defatted seeds to a fine powder, the defatted flour was seived through a 100 mesh-sieve, and stored in a sealed plastic bag until use.

### 3.2. Alkaline Treatment

A defatted rambutan seed flour sample (100 g) was suspended in NaOH solution (1 L, 0.3% *w*/*v*) under constant stirring for 4 h. The suspension was centrifuged at 5000 rpm for 30 min, and the supernatant was discarded. The slurry was mixed with distilled water and centrifuged again. The washing procedure was repeated five times keeping pH 8.0–9.0. The remaining sediment was oven-dried overnight at 50 °C, ground and then screened using a 100 mesh-sieve. It was stored in a sealed plastic bag until use.

### 3.3. Chemical Composition

Moisture, fat, protein and ash contents were determined following the AOAC methods [12]. Amylose content was determined in compliance with the guidelines described in the Thai Agricultural Standard (TAS) number 4000-2003 on Thai Hom Mali rice [13].

### 3.4. Color and Turbidity

The color of samples were measured using a Chroma Meter (Model CR-400, Konica Minolta, Osaka, Japan). Turbidity was determined by measuring the absorbance of suspension (2% *w*/*v*), of which was adjusted the suspension to pH 7.0, at 640 nm against a water blank with a Jenway UV-visible spectrophotometer (Model 6405, Jenway Limited, Essex, UK).

### 3.5. Bulk Density, Water and Oil Absorption Capacity

Bulk density was determined using a 10 mL graduated cylinder and a 1 g sample. The cylinder was carefully tapped until each sample was leveled out. The bulk density was expressed as g/mL. For water absorption, suspensions (10% *w*/*v*) were prepared with distilled water and then centrifuged. The supernatant was discarded, and the wet sediment was weighed. Similarly for the determination of oil absorption, grape seed oil as a representative of high content unsaturated fatty acids was used. The water and oil adsorption were determined as g of water or oil adsorbed per g of the sample on a dry-weight basis.

### 3.6. Solubility, Swelling Power and Emulsion Properties

Suspensions (1% *w*/*v*) were first prepared with distilled water, heated to 85 °C for 30 min, followed by centrifugation at 5000 rpm for 30 min. The supernatant was transferrred into an aluminium can and dried at 105 °C in an oven overnight. The wet sediment was weighed. Solubility was calculated as the weight of dried supernatant divided by the initial weight of dry sample, reported as g/100 g. Swelling power was defined as the weight of wet sediment to the initial weight of dry sample.

To determine emulsion properties, samples (1.0 g) were suspended in distilled water (6 mL) and grape seed oil (6 mL) was added. The dispersed samples were mixed using a vortex mixer for 5 min and then centrifuged at 5000 rpm for 30 min. Emulsion capacity was the ratio of the volume of emulsified layer to the whole emulsion volume.

After preparation of the test samples, they were homogenized at 3400 rpm for 2 min, heated to 85 °C for 15 min, followed by centrifugation as previously described. The emulsion stability was determined as the volume of emulsified layer to that of the heated emulsion.

### 3.7. Pasting Properties

Pasting properties of samples were determined using a rapid visco analyzer (RVA-TecMaster, Newport Scientific, New South Wales, Australia) with a paddle rotating at a constant 160 rpm. Sample dispersions of 16 g/100 mL were equilibrated at 50 °C for 1 min, heated from 50 to 95 °C in 5 min, maintained at 95 °C for 2.5 min, cooled to 50 °C in 4 min and held at 50 °C for 2 min. Pasting parameters such as the pasting temperature, peak time, final viscosity, breakdown and setback were obtained.

### 3.8. Least Gelation Concentration

Suspensions of 2%, 4%, 6%, 8%, 10%, 12%, 14% and 16% (*w*/*v*) were prepared in test tubes with 5 mL distilled water, heated for 1 h at 95 °C in a water bath, followed by cooling to 10 °C. The gelation results were expressed as no (−), complete (+) or partial (±) gelling and the least gelation concentration was the lowest value at which a complete gelling occurred.

### 3.9. Apparent Viscosity

Samples of 5%, 10%, 15%, 20% and 25% (*w*/*v*) were prepared with distilled water. All samples were measured for apparent viscosity at 27 ± 1 °C using a Brookfield DV-III Ultra viscometer (Brookfield Engineering Laboratories Inc., Middleboro, MA, USA) with a T-bar spindle at a rotating speed of 100 rpm.

### 3.10. Statistical Analysis

The experiments were repeated at least two times for each SC-CO_2_ extraction. All measurements were performed at least twice using the prepared samples and were reported as calculated means and standard deviations. Data were statistically analyzed using the SPSS software (version 13.0, SPSS Inc., Chicago, IL, USA). Differences were considered significant at *p* < 0.05.

## 4. Conclusions

Our results show that the physicochemical properties of defatted rambutan seed flour were affected by alkali treatment. The overall changes were an increase in bulk density, swelling power, water absorption capacity, emulsion capacity and stability, but a decrease in turbidity, solubility, and oil absorption capacity. Alkali-treated flour showed a significant increase in peak viscosity, breakdown, setback and final viscosity with reduction in pasting temperature. The alkali treatment resulted in a decrease in the least gelation concentration, but an increase in apparent viscosity. This study presents preliminary data for further investigations on rambutan seed starch.

## Figures and Tables

**Table 1:** Chemical composition (g/100 g) for untreated and alkali-treated defatted rambutan seed flours.

Sample	Protein	Fat	Ash	Amylose
Untreated	10.46 ± 0.05 ^a^	6.64 ± 0.31 ^a^	1.54 ± 0.01 ^b^	19.09 ± 0.04 ^a^
Treated	9.50 ± 1.01 ^b^	4.99 ± 0.01 ^b^	1.87 ± 0.13 ^a^	17.94 ± 0.02 ^b^

Means with different letters in same column differ significantly (*p* < 0.05), *n* = 2.

**Table 2:** Hunter color values (L*, a*, b*), chroma and turbidity for untreated and alkali-treated defatted rambutan seed flours.

Sample	Hunter Color Values			Chroma	Turbidity
	L*	a*	b*		(ABS)
Untreated	92.14 ± 0.24 ^a^	4.09 ± 0.04 ^b^	9.26 ± 0.12 ^b^	10.13 ± 0.10 ^b^	2.78 ± 0.01 ^a^
Treated	90.26 ± 0.16 ^b^	4.84 ± 0.01 ^a^	11.14 ± 0.32 ^a^	12.15 ± 0.29 ^a^	2.11 ± 0.01 ^b^

Means with different letters in same column differ significantly (*p* < 0.05), *n* = 3.

**Table 3:** Bulk density, water and oil absorption capacity of untreated and alkali-treated defatted rambutan seed flours.

Sample	Bulk Density	Water Absorption	Oil Absorption
	(g/mL)	(g/g)	(g/g)
Untreated	0.36 ± 0.01 ^b^	2.56 ± 0.01 ^b^	1.41 ± 0.04 ^a^
Treated	0.65 ± 0.01 ^a^	3.90 ± 0.04 ^a^	1.25 ± 0.05 ^b^

Means with different letters in same column differ significantly (*p* < 0.05), *n* = 3.

**Table 4:** Viscosity parameters of untreated and alkali-treated defatted rambutan seed flours.

Sample	Pasting	Peak Time	Peak	Breakdown	Final	Setback
	Temperature (°C)	(min)	Viscosity (cP)	(cP)	Viscosity (cP)	(cP)
Untreated	89	7	1056	86	1244	275
Treated	68	6	3055	647	4050	1643

Determinations carried out in triplicates.

**Table 5:** Solubility, swelling power and emulsion properties of untreated and alkali-treated defatted rambutan seed flours.

Sample	Solubility	Swelling Power	Emulsion Properties	
	(g/100 g)	(g/g)	Capacity (mL/100 mL)	Stability (mL/100 mL)
Untreated	17.69 ± 0.31 ^a^	10.64 ± 0.20 ^b^	47.69 ± 1.54 ^b^	34.55 ± 1.38 ^b^
Treated	13.99 ± 0.78 ^b^	13.84 ± 0.68 ^a^	61.22 ± 1.94 ^a^	51.79 ± 0.89 ^a^

Means with different letters in same column differ significantly (*p* < 0.05), *n* = 3.

**Table 6:** The least gelation concentrations of untreated and alkali-treated defatted rambutan seed flours.

Sample	Concentration (g/100 mL)							
	2	4	6	8	10	12	14	16
Untreated	−	−	−	−	±	±	+	+
Treated	±	±	±	+	+	+	+	+

Determinations carried out in duplicates. No gelling (−), complete gelling (+) or partial gelling (±).
